# Assessing the Prognostic Value of Preoperative Carcinoembryonic Antigen-Specific T-Cell Responses in Colorectal Cancer

**DOI:** 10.1093/jnci/djv001

**Published:** 2015-02-10

**Authors:** Martin J. Scurr, Clare M. Brown, Diana F. Costa Bento, Gareth J. Betts, Brian I. Rees, Robert K. Hills, Awen Gallimore, Andrew Godkin

**Affiliations:** Affiliations of authors:Institute of Infection and Immunity, School of Medicine, Cardiff University, Cardiff, UK (MJS, CMB, DFCB, AwG, AnG); Nuffield Department of Surgical Sciences, Oxford University, John Radcliffe Hospital, Oxford, UK (GJB); Department of Colorectal Surgery, University Hospital of Wales, Cardiff, UK (BIR); Cancer Trials Unit (Translational Statistics), School of Medicine, Cardiff University, Cardiff, UK (RKH); Department of Integrated Medicine, University Hospital of Wales, Cardiff, UK (AnG).

## Abstract

Current dogma suggests that tumor-reactive IFN-γ–producing (T_H_1-type) T-cells are beneficial to patient outcome; however, the clinical consequence of these responses with respect to long-term prognosis in colorectal cancer (CRC) is not understood. Here, we compared the utility of preoperative, peripheral blood–derived IFN-γ^+^ T-cell responses specific to carcinoembryonic antigen (CEA), 5T4, or control antigens (n = 64) with tumor staging and clinical details (n = 87) in predicting five-year outcome of CRC patients who underwent resection with curative intent. Although disease recurrence was more likely in patients with stage III tumors, the presence of preoperative, CEA-specific IFN-γ–producing T-cells identified patients at a statistically significantly greater risk of tumor recurrence following surgical resection, irrespective of tumor stage (odds ratio = 5.00, 95% confidence interval = 1.96 to 12.77, two-sided *P* <.001). Responses to other antigens, including 5T4, did not reflect outcome. Whilst these results initially appear surprising, they could improve prognostication and help redirect adjuvant treatments.

Colorectal cancer (CRC) accounts for more than 600 000 deaths per year worldwide (1). Where possible, resection of the primary tumor is performed with curative intent; however, 40% to 50% of these patients will relapse or die from metastatic disease (2). The current benchmark for predicting survival and guiding treatment is clinicopathological staging of the excised tumor. Patients with a Tumor-Node-Metastasis (TNM) stage I tumor have a predicted five-year survival of greater than 90%; stage II tumor, approximately 70% to 85%; stage III tumor, approximately 40% to 55%; and stage IV tumor, less than 10% (3).

T_H_1-type (IFN-γ–producing) CD4^+^ and CD8^+^ T-cells, which recognize tumor antigens, may play a critical role in antitumor immunity, as illustrated by adoptive transfer of T-cells into cancer-bearing recipients (4–6). The presence of tumor-specific T-cells is not always conducive with beneficial outcome: An increased pretreatment frequency of MART1- and NY-Eso-1–specific CD8^+^ T cells in melanoma patients receiving anti–PD-1 treatment was associated with a poorer outcome. Whether these T-cells produced IFN-γ is not clear (7). We have previously identified peripheral blood-derived T-cell responses in preoperative CRC patients specific to the tumor-associated antigens carcinoembryonic antigen (CEA), an autoantigen expressed at low levels in normal intestinal epithelia (8,9), and 5T4, an oncofetal antigen that is not expressed in healthy adult tissues (10,11). Both proteins are markedly upregulated in most CRCs, however the clinical significance of tumor-specific vs autoantigen-specific T-cell responses with respect to long-term prognosis is not understood. For some individuals, these responses are only measurable after in vitro depletion of regulatory T-cells (Tregs), which suppress antitumor immunity (10,12,13).

Here, we present the clinico-demographic details of 87 patients, recruited to a single center, undergoing an intended curative resection of primary CRC (TNM stage I-III). Patients gave written informed consent and were followed for up to five years (clinical details shown in Supplementary Table 1, available online); the Cardiff and Vale University ethics committee granted approval for this study. Immune responses were measured in 64 patients, where blood was available for experiments, allowing an analysis of whether immunological characteristics correspond to outcome. IFN-γ–producing T-cell responses to tumor antigens (CEA, 5T4) and control antigens (PPD, HA) were enumerated by ex vivo ELISpot as described (10,12). Briefly, unmanipulated or CD25^hi^CD4^+^ (>90% Foxp3^+^) Treg-depleted peripheral blood mononuclear cells were stimulated with either 1 μg/mL CEA, 5T4, PPD, HA, or media alone, in anti-IFN-γ–coated ELISpot plates (MabTech AB, Sweden). Following incubation, plates were developed and spots (ie, activated antigen-specific T-cells) counted using an automated plate reader (A.I.D. GmbH, Germany); positive responses were defined as described (Supplementary Figure 1, available online). All statistical tests were two-sided, and a *P* value of less than .05 was considered statistically significant.

During the five-year follow-up period, 33 patients died: 25 because of CRC, five because of unrelated causes, and three because of unknown causes. There was little difference (*P* = .38) between overall and disease-free survival (Supplementary Figure 2A, available online), indicating that tumor recurrence, determined by clinical examination and imaging (CT and/or colonoscopy), was mainly responsible for mortality. As expected, likelihood of five-year tumor recurrence steadily increased from TNM stage I (18.2%), stage II (33.3%) to stage III tumors (51.4%), with a statistically significant increase in five-year tumor recurrence in stage III patients (hazard ratio [HR] = 2.43, 95% confidence interval [CI] =1.12 to 5.30, two-sided log-rank test *P* = .04) (Supplementary Figure 2B, available online). Age, anatomical location of the tumor, and gender did not influence the rate of recurrence as determined by univariate and multivariable analyses (*P* = .99, .29, and .44, respectively) (Supplementary Figure 2, C-F, available online).

IFN-γ^+^ T-cell responses of different antigen specificity were associated with five-year patient survival. The Kaplan-Meier curves (Figure 1, A-D) reveal that the presence or absence of CEA-specific T_H_1 responses, identified either before or after Treg depletion, statistically significantly distinguish patients most likely to suffer tumor recurrence, even at an early time point (log-rank test *P* = .002). In contrast, the presence or absence of responses to 5T4 (*P* = .98) and the control antigens PPD (*P* = .66) and HA (*P* = .84) did not associate with tumor recurrence. However, removing 5T4-responsive patients from those CEA-responders reveals a worsening of survival (*P* < .001), suggesting benefit in anti-5T4 immune responses in this subgroup (Figure 1, E-F).

**Figure 1.**
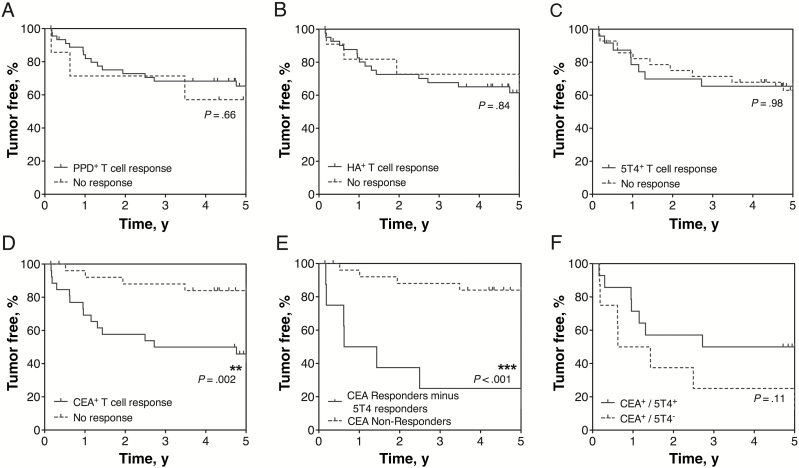
Tumor status compared with preoperative T-cell responses to four distinct antigens. T-cell responses were measured by ex vivo IFN-γ ELISpot to the control antigens tuberculin purified protein derivative (PPD) **(A)**, haemagglutinin (HA) **(B)**, and the tumor antigens 5T4 **(C)** and CEA **(D)**, before and after the depletion of CD4^+^CD25^hi^ regulatory T cells from freshly isolated peripheral blood mononuclear cells. Patients were grouped according to the presence or absence of a measured T-cell response, and this was associated with survival over five years following initial tumor resection. The outcome of patients who did not produce a measureable T-cell response to CEA, either before or after Treg depletion, was compared with those patients who did mount an anti-CEA T-cell response, minus the patients who also responded to 5T4 **(E)**. The effect of producing an anti-5T4 T-cell response amongst CEA responders was also associated with survival **(F)**. Statistically significant differences are indicated (**P < .01, ***P < .001). All statistical tests were two-sided. For the numbers of patients at risk for each group in the curves, please see Supplementary Table 2 (available online). CEA = carcinoembryonic antigen; PPD = purified protein derivative.

The association of CEA-specific T-cell responses and poor outcome remains when patients are stratified by tumor stage (Figure 2A). Strikingly, tumor recurrence was more likely in stage I/II patients with a CEA-specific response than stage III patients with no CEA-specific response. When these responses were further stratified on a Forest plot, there was a consistency of effect over all tumor stages (Figure 2B: odds ratio [OR] = 5.00, 95% CI = 1.96 to 12.77, *P* < .001). Despite limited power, tests for trend and heterogeneity revealed no evidence of heterogeneity of effect by tumor stage (*P* = 1.0). Furthermore, in a multivariable Cox model, the quantitative measure of each patients’ CEA^+^ T-cell response was the most powerful prognostic factor when assessing all covariables (*P* = .004 for entry); after adjustment for this in the model, only TNM status was statistically significant at *P* < .05 (Supplementary Figure 2F, available online). Assumptions of proportionality were determined and satisfied by examination of log (-log) survival vs log (time) plots (data not shown). In a model with these two variables fitted and using bootstrapping with 1000 iterations, the hazard ratio for CEA^+^ T-cell response was 1.04 (95% Wald CI = 1.02 to 1.06) and for the hazard ratio for TNM status was 2.30 (95% Wald CI = 1.07 to 4.96) (data not shown, results from the bootstrapping analysis). Consistent with the results of Figure 2, A and B, whilst power is limited, there was no evidence of interaction between CEA response and TNM status (*P* = .99) (data not shown). These data strongly imply that the presence (and to a lesser degree magnitude) of CEA-specific T-cell responses preoperatively are detrimental to postsurgical outcome and are far more statistically significant than tumor stage in identifying patients with disease recurrence.

**Figure 2.**
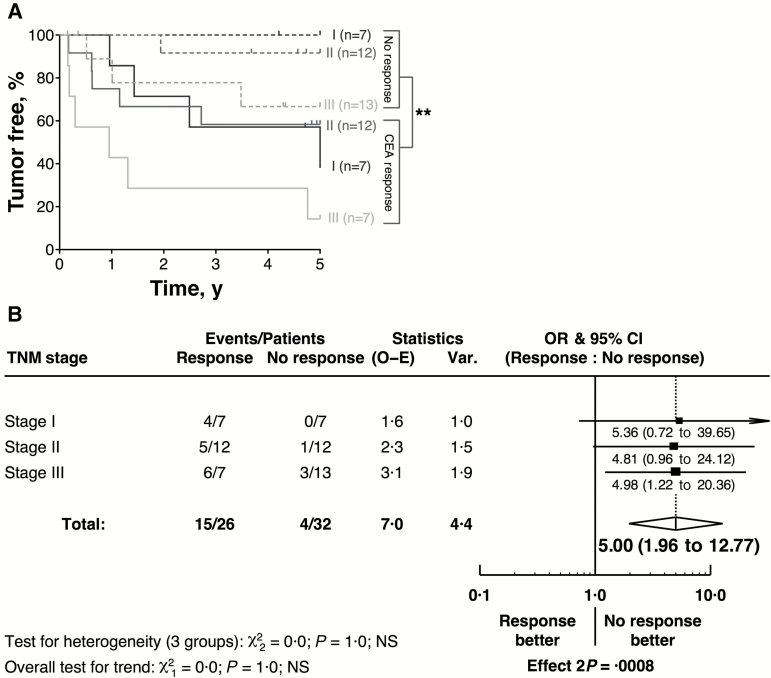
CEA-specific T-cell responses and tumor recurrence. Irrespective of the tumor stage, patients producing an anti-CEA IFN-γ^+^ T-cell response were more likely to suffer a tumor recurrence, as demonstrated in the Kaplan-Meier curve **(A)** and the Forest plot **(B)**. The statistically significant difference is indicated (**P < .01). All statistical tests were two-sided. For the numbers of patients at risk for each group in the curves please see Supplementary Table 2 (available online). CEA = carcinoembryonic antigen; CI = confidence interval; OR = odds ratio; TNM = Tumor-Node-Metastasis.

Currently there is much interest in targeting CEA via anticancer vaccination or T-cell–directed adoptive transfer (14,15), thus our findings open up debate about the potential risks of such strategies in certain situations. Why should CEA-specific, but not 5T4-specific, responses be detrimental? There are data demonstrating that adoptive transfer of CEA-specific T-cells to control CRC induces enteropathy in normal colonic tissue (16,17). It is possible that a loss of mucosal integrity with increased epithelial leakiness facilitates tumor growth or recurrence (18,19).

Additionally, upon examination of primary tumor samples from the cohort described herein, intratumoral T-cells were dominated by the expression of the transcription factor RORγT^+^, a marker for T_H_17 cells; however, this preponderance of RORγT^+^ T-cells within tumors was found in both CEA responders and nonresponders (Supplementary Figure 3, available online). The dominance of T_H_17 type cells in all patient groups is unexpected. We have optimized peripheral blood assays for measuring IL-17 responses, but as part of an ongoing study in a new group of CRC patients, peripheral blood–derived CEA-specific IL-17–producing T-cells are observed in less than 5% of patients (data not shown). The relationship between blood-derived and tumor-derived antigen-specific T-cell responses awaits further investigation.

CEA-specific T-cell responsiveness could also reflect more advanced tumor stages, ie, presence of micrometastases that are not demonstrated by conventional staging techniques. Whilst further studies are required to uncover why CEA-specific T-cells confer harm, it is clear that measuring these responses could offer crucial prognostic information independent of tumor stage and help identify patients at risk for tumor recurrence. Although this particular study was limited by the relatively small sample size without a validation cohort and by the fact that immunological data were not available for all patients, the conclusions from this study raise the possibility that targeting adjuvant treatments at CEA^+^ T-cell responders may improve treatment outcome.

## Funding

This work was supported by Cancer Research Wales, Tenovus, the Wellcome Trust (grant number WT086983), and the Medical Research Council (grant number G0801190).

## Supplementary Material

Supplementary Data

## Notes

The study funders had no role in the design of the study, the collection, analysis, or interpretation of the data, the writing of the manuscript, nor the decision to submit the manuscript for publication.

The authors are grateful to Dr. Gary Reynolds (University of Birmingham) for constructive assistance with immunohistochemistry and Peter Scurr (Bank of Ireland) for statistical advice.

## References

[1] Jemal A Bray F Center MM Global cancer statistics. CA Cancer J Clin. 2011;61(2):69–90. 21296855 10.3322/caac.20107

[2] Meyerhardt JA Mayer RJ Systemic therapy for colorectal cancer. N Engl J Med. 2005;352(5):476–487. 15689586 10.1056/NEJMra040958

[3] O’Connell JB Maggard MA Ko CY Colon cancer survival rates with the new American Joint Committee on cancer sixth edition staging. J Natl Cancer Inst. 2004;96(19):1420–1425. 15467030 10.1093/jnci/djh275

[4] Hunder NN Wallen H Cao J Treatment of metastatic melanoma with autologous CD4+ T cells against NY-ESO-1. N Engl J Med. 2008;358(25):2698–2703. 18565862 10.1056/NEJMoa0800251 PMC3277288

[5] Chapuis AG Thompson JA Margolin KA Transferred melanoma-specific CD8+ T cells persist, mediate tumor regression, and acquire central memory phenotype. Proc Natl Acad Sci U S A. 2012;109(12):4592–4597. 22393002 10.1073/pnas.1113748109 PMC3311364

[6] Weide B Zelba H Derhovanessian E Functional T cells targeting NY-ESO-1 or Melan-A are predictive for survival of patients with distant melanoma metastasis. J Clin Oncol. 2012;30(15):1835–1841. 22529253 10.1200/JCO.2011.40.2271

[7] Weber JS Kudchadkar RR Yu B Safety, efficacy, and biomarkers of nivolumab with vaccine in ipilimumab-refractory or naïve melanoma. J Clin Oncol. 2013;31(34):4311–4318. 24145345 10.1200/JCO.2013.51.4802 PMC3837092

[8] Davidson BR Sams VR Styles J Comparative study of carcinoembryonic antigen and epithelial membrane antigen expression in normal colon, adenomas and adenocarcinomas of the colon and rectum. Gut. 1989;30(9):1260–1265. 2806995 10.1136/gut.30.9.1260 PMC1434233

[9] Hammarstrom S The carcinoembryonic antigen (CEA) family: structures, suggested functions and expression in normal and malignant tissues. Semin Cancer Biol. 1999;9(2):67–81. 10202129 10.1006/scbi.1998.0119

[10] Betts G Jones E Junaid S Suppression of tumour-specific CD4(+) T cells by regulatory T cells is associated with progression of human colorectal cancer. Gut. 2012;61(8):1163–1171. 22207629 10.1136/gutjnl-2011-300970 PMC3388728

[11] Southall PJ Boxer GM Bagshawe KD Immunohistological distribution of 5T4 antigen in normal and malignant tissues. Br J Cancer. 1990;61(1):89–95. 2404511 10.1038/bjc.1990.20 PMC1971328

[12] Clarke SL Betts GJ Plant A CD4+CD25+FOXP3+ regulatory T cells suppress anti-tumor immune responses in patients with colorectal cancer. PloS ONE. 2006;1:e129. 17205133 10.1371/journal.pone.0000129 PMC1762416

[13] Nishikawa H Sakaguchi S Regulatory T cells in tumor immunity. Int J Cancer. 2010;127(4):759–767. 20518016 10.1002/ijc.25429

[14] Turriziani M Fantini M Benvenuto M Carcinoembryonic antigen (CEA)-based cancer vaccines: recent patents and antitumor effects from experimental models to clinical trials. Recent Pat Anticancer Drug Discov. 2012;7(3):265–296. 22630596 10.2174/157489212801820020

[15] Gameiro SR Jammeh ML Hodge JW Cancer vaccines targeting carcinoembryonic antigen: state-of-the-art and future promise. Expert Rev Vaccines. 2013;12(6):617–629. 23750792 10.1586/erv.13.40

[16] Bos R van Duikeren S Morreau H Balancing between antitumor efficacy and autoimmune pathology in T-cell-mediated targeting of carcinoembryonic antigen. Cancer Res. 2008;68(20):8446–8455. 18922918 10.1158/0008-5472.CAN-08-1864

[17] Parkhurst MR Yang JC Langan RC T cells targeting carcinoembryonic antigen can mediate regression of metastatic colorectal cancer but induce severe transient colitis. Mol Ther. 2011;19(3):620–626. 21157437 10.1038/mt.2010.272 PMC3048186

[18] Grivennikov SI Wang K Mucida D Adenoma-linked barrier defects and microbial products drive IL-23/IL-17-mediated tumour growth. Nature. 2012;491(7423):254–258. 23034650 10.1038/nature11465 PMC3601659

[19] Gallimore AM Godkin A Epithelial barriers, microbiota, and colorectal cancer. N Engl J Med. 2013;368(3):282–284. 23323906 10.1056/NEJMcibr1212341

