# Supplementary material for: Assessing the Prognostic Value of Preoperative Carcinoembryonic Antigen-Specific T-Cell Responses in Colorectal Cancer
Source: J Natl Cancer Inst. 2015 Feb 10;107(4):djv001. doi: 10.1093/jnci/djv001 (PMC4394893; doi:10.1093/jnci/djv001)
Supplement: Supplementary Data [file supp_107_4_djv001__index.html]

Assessing the Prognostic Value of Preoperative Carcinoembryonic Antigen-Specific T-Cell Responses in Colorectal Cancer — Supplementary Data 

# Assessing the Prognostic Value of Preoperative Carcinoembryonic Antigen-Specific T-Cell Responses in Colorectal Cancer

## Supplementary Data

Data files

**Files in this Data Supplement:**

- Supplementary Data - Supplementary Data
