# Supplementary material for: Assessing the Prognostic Value of Preoperative Carcinoembryonic Antigen-Specific T-Cell Responses in Colorectal Cancer
Source: J Natl Cancer Inst. 2015 Feb 10;107(4):djv001. doi: 10.1093/jnci/djv001 (PMC4394893; doi:10.1093/jnci/djv001)
Supplement: Supplementary Data [file supp_djv001_jnci_JNCI_14_1049_s01.docx]

**Supplementary Table 1:** Characteristics of the colorectal cancer patients included in this study.

| **Patients** | **n = 87** |
| --- | --- |
| **Male : Female** | 53 : 34 |
| **Age Range, y** | 30-96 |
| **Tumor Location, No. (%)** |  |
| Ascending / Transverse | 27 (31) |
| Descending / Sigmoid | 32 (27) |
| Rectum | 28 (32) |
| **TNM Stage, 5th Edition, No. (%)** |  |
| I | 16 (18) |
| II | 32 (37) |
| III | 39 (45) |

**Supplementary Table 2:** Number of patients at risk for all Kaplan-Meier Curves

| **Number of patients at risk** | |  |  |  |  |  |  |  |
| --- | --- | --- | --- | --- | --- | --- | --- | --- |
| **Figure** | **Sub-Group** | **Years** |  |  |  |  |  |  |
|  |  | **0** | **1** | **2** | **3** | **4** | **5** |  |
| **1A** | PPD+ T cell response | 47 | 37 | 32 | 30 | 29 | 21 |  |
|  | No response | 8 | 5 | 5 | 5 | 4 | 2 |  |
| **1B** | HA+ T cell response | 44 | 33 | 29 | 27 | 25 | 15 |  |
|  | No response | 11 | 9 | 8 | 8 | 8 | 8 |  |
| **1C** | 5T4+ T cell response | 24 | 18 | 16 | 15 | 15 | 11 |  |
|  | No response | 31 | 24 | 21 | 20 | 18 | 12 |  |
| **1D** | CEA+ T cell response | 26 | 18 | 15 | 13 | 13 | 9 |  |
|  | No response | 29 | 24 | 22 | 22 | 20 | 18 |  |
| **1E** | CEA responders minus 5T4 responders | 8 | 4 | 3 | 2 | 2 | 2 |  |
|  | CEA Non-Responders | 29 | 24 | 22 | 22 | 20 | 18 |  |
| **1F** | CEA+/5T4+ | 14 | 10 | 8 | 7 | 7 | 4 |  |
|  | CEA+/5T4- | 8 | 4 | 3 | 2 | 2 | 2 |  |
| **2A** | No response Stage I | 7 | 7 | 7 | 7 | 7 | 5 |  |
|  | No response Stage II | 12 | 12 | 11 | 11 | 10 | 8 |  |
|  | No response Stage III | 13 | 8 | 7 | 7 | 6 | 4 |  |
|  | CEA response Stage I | 7 | 6 | 5 | 5 | 4 | 3 |  |
|  | CEA response Stage II | 12 | 9 | 8 | 7 | 7 | 5 |  |
|  | CEA response Stage III | 7 | 3 | 2 | 2 | 2 | 1 |  |
| **S. 2A** | Overall Survival | 87 | 71 | 59 | 53 | 48 | 36 |  |
|  | Disease Free Survival | 87 | 62 | 52 | 48 | 45 | 34 |  |
| **S. 2B** | TNM Stage I | 16 | 14 | 13 | 12 | 12 | 9 |  |
|  | TNM Stage II | 32 | 27 | 24 | 21 | 21 | 17 |  |
|  | TNM Stage III | 39 | 26 | 18 | 18 | 15 | 11 |  |
| **S. 2C** | 30-49 | 5 | 3 | 3 | 3 | 3 | 2 |  |
|  | 50-59 | 12 | 11 | 9 | 8 | 8 | 7 |  |
|  | 60-69 | 26 | 16 | 16 | 15 | 13 | 8 |  |
|  | 70-79 | 21 | 15 | 14 | 12 | 11 | 9 |  |
|  | 80-99 | 23 | 17 | 10 | 10 | 10 | 8 |  |
| **S. 2D** | Ascending / Transverse Colon | 27 | 19 | 13 | 11 | 10 | 6 |  |
|  | Descending / Sigmoid Colon | 32 | 25 | 23 | 23 | 21 | 16 |  |
|  | Rectum | 28 | 18 | 16 | 14 | 14 | 12 |  |
| **S. 2E** | Female | 34 | 25 | 21 | 20 | 19 | 14 |  |
|  | Male | 53 | 37 | 31 | 28 | 26 | 20 |  |
|  |  |  |  |  |  |  |  |  |
|  |  |  |  |  |  |  |  |  |

**Supplementary Figure 1** Schematic demonstrating how IFN-γ ELISpots were carried out and analysed (A). Examples of a non-responder (patient 6), a responder (patient 65) and a response that could only be detected after depletion of CD4^+^CD25^hi^ cells (patient 35).

**Supplementary Figure 2** Summary of the proportion of patients alive at each time point (overall survival), and the proportion of patients alive and tumor free at each time point (disease-free survival) (A). Kaplan-Meier curves indicating the time to tumor recurrence in patients grouped according to a series of clinical characteristics: (B) histopathological tumor stage, (C) age, (D) anatomical location of the primary tumor and (E) gender. Significant difference is indicated (* P<0.05). To assess the independent prognostic factors for survival, Cox regression was performed using age, gender, CRC location, TNM stage and quantitative measures of IFN-γ^+^ T-cell responses to 5T4 and CEA as candidate variables, with forward selection and an entry probability of *P*=0.004. After adjustment for CEA response, only TNM stage was statistically significant at *P*<0.05 (*P*>0.3 for all other covariates) (F). For the numbers of patients at risk for each group in the curves please see **Supplementary Table 2**.

**Supplementary Figure 3** Colorectal tumor sections were stained with anti-CD3, -T-bet and -RORγt antibodies. Representative images from CD3^+^ (Gray) / T-bet^+^ (Brown) (A), and CD3^+^ (Gray) / RORγt^+^ (Brown) T cells (B), with examples of counted cells shown by green arrows. T_H_1 (CD3^+^T-bet^+^) and T_H_17 (CD3^+^RORγt^+^) cells were enumerated in CEA responders (n=5) and non-responders (n=7) (C), and the T_H_17: T_H_1 ratio determined (D). One high power field of view is equivalent to 600x magnification. The result of an unpaired, two-sided t--test to compare the two groups is shown.
